# Supplementary material for: The awareness of novelty for strangely familiar words: a laboratory analogue of the déjà vu experience
Source: PeerJ. 2014 Nov 11;2:e666. doi: 10.7717/peerj.666 (PMC4230551; doi:10.7717/peerj.666)
Supplement: Appendix S1 — Within list type, upper-case N indicates high novelty lists, lower-case n indicates low novelty lists. Upper-case F indicates high familiarity lists, lower-case f indicates low familiarity lists. Test words are followed by their stimulus type in parentheses. cl, critical lure; rl, related lure; ul, related lure; t, target. [file peerj-02-666-s001.docx]

| **list type** | | N/F | N/f | n/F | n/f |
| --- | --- | --- | --- | --- | --- |
| **string** | | SM | SHI | TA | W |
| **list** | **position** |  |  |  |  |
| study | 1 | lungs | collar | rocking | ink |
|  | 2 | cigarette | tie | cushion | white |
|  | 3 | chimney | pocket | sit | brown |
|  | 4 | cigar | blouse | wood | blue |
|  | 5 | stink | shorts | seat | charred |
|  | 6 | stain | vest | sitting | coal |
|  | 7 | billows | jersey | stool | death |
|  | 8 | pollution | sleeves | bench | funeral |
|  | 9 | pipe | button | sofa | grief |
|  | 10 | blaze | iron | legs | bottom |
|  | 11 | ashes | linen | desk | cat |
|  | 12 | tobacco | polo | table | night |
| test | 1 | pipe (t) | linen (t) | rocking (t) | ink (t) |
|  | 2 | flames (rl) | piano (ul) | helmet (ul) | cauliflower (ul) |
|  | 3 | lungs (t) | pants (rl) | poem (ul) | coal (t) |
|  | 4 | puff (rl) | voltage (ul) | couch (rl) | funeral (t) |
|  | 5 | jewel (ul) | tie (t) | recliner (rl) | grey (rl) |
|  | 6 | stain (t) | collar (t) | chair (cl) | color (rl) |
|  | 7 | smoke (cl) | shirt (cl) | wood (t) | thermometer (ul) |
|  | 8 | mathematics (ul) | belt (rl) | sofa (t) | black (cl) |
